# 

**Published:** 1875-04

**Authors:** 


### Books and Pamphlets Received.

A Manual of Diet in Health and Disease. By Thomas King Chambers, M. D., Oxon., F. R. C. P., London. Philadelphia: Henry C. Lea, 1875. Buffalo: T. Butler & Son.

A Series of American Clinical Lectures. Edited by E. C. Seguin, M. D. Vol. I. No. III. Pneumo-Thorax. By Austin Flint, Sr., M. D. New York: G. P. Putnam's Sons, 1875.

The History of the Philadelphia School of Anatomy and its relations to Medical Teaching. A Lecture delivered March 1, 1875, at its dissolution. By W. W. Keen, M. D., Lect. on Anatomy and Operative Surgery. Philadelphia: J. B. Lippincott & Co., 1875. Buffalo: T. Butler & Son.

Reports of the Trustees and Superintendent of the Butler Hospital for the Insane, Providence, R. I.

Proceedings of the Royal Society, London. Vol. XXII. Nos. 148–155; Vol. XXIII. No. 156.

### Medical and Surgical Journal

PUBLISHED MONTHLY,

**Containing Original Articles, Reports of Medical Societies and Hospitals, Editorial, Reviews, Correspondence, Army News, etc., etc; including the usual variety of Medical Periodical Publications. Specimen copies sent on application.**

Address Buffalo Medical & Surgical Journal, Buffalo, N. Y.

TERMS OF SUBSCRIPTION, - - $3.00 PER YEAR, IN ADVANCE.

